# Chasing the signaling run by tri-molecular time-lapse FRET microscopy

**DOI:** 10.1038/s41420-018-0047-4

**Published:** 2018-03-22

**Authors:** Hsiang-Ling Kuo, Pei-Chuan Ho, Shenq-Shyang Huang, Nan-Shan Chang

**Affiliations:** 1 Institute of Molecular Medicine, National Cheng Kung University, Tainan, Taiwan Republic of China; 2 Center of Infectious Disease and Signaling Research, National Cheng Kung University, Tainan, Taiwan Republic of China; 3 Advanced Optoelectronic Technology Center, National Cheng Kung University, Tainan, Taiwan Republic of China; 4 Department of Neurochemistry, New York State Institute for Basic Research in Developmental Disabilities, Staten Island, NY USA; 5 Graduate Institute of Biomedical Sciences, College of Medicine, China Medical University, Taichung, Taiwan Republic of China; 6 Present Address: Graduate Program of Biotechnology in Medicine, Institute of Molecular and Cellular Biology, National Tsing Hua University, Hsinchu, Taiwan Republic of China

## Abstract

A feasible design is made to measure three protein/protein interactions to visualize signal pathways by time-lapse Förster resonance energy transfer (FRET) microscopy. When interacting proteins are in close proximity, excitation energy is provided to allow the energy flow from the first molecule to excite the second, followed by energy transfer to the third. By phorbol ester/calcium ionophore stimulation, for example, a real-time complex formation of ectopic IκBα/ERK/WWOX occurs as measured by FRET microscopy, indicative of an ongoing functional signaling. Hyaluronan induces membrane Hyal-2 signaling, which allows FRET measurement of the complex formation of ectopic Smad4/WWOX/Hyal-2 for causing bubbling cell death. If ectopic p53 is recruited to replace Hyal-2, the resulting ectopic Smad4/WWOX/p53 complex induces membrane blebbing without cell death. Together, in this perspective review article, we demonstrate the utilization of time-lapse FRET microscopy to visualize the signaling event via the tri-molecular protein complex formation and their biological outcomes. We show an initial two-protein binding to form the driving force to jumpstart the tri-molecular execution for the signal pathway.

## Introduction

**Fig. 1:**
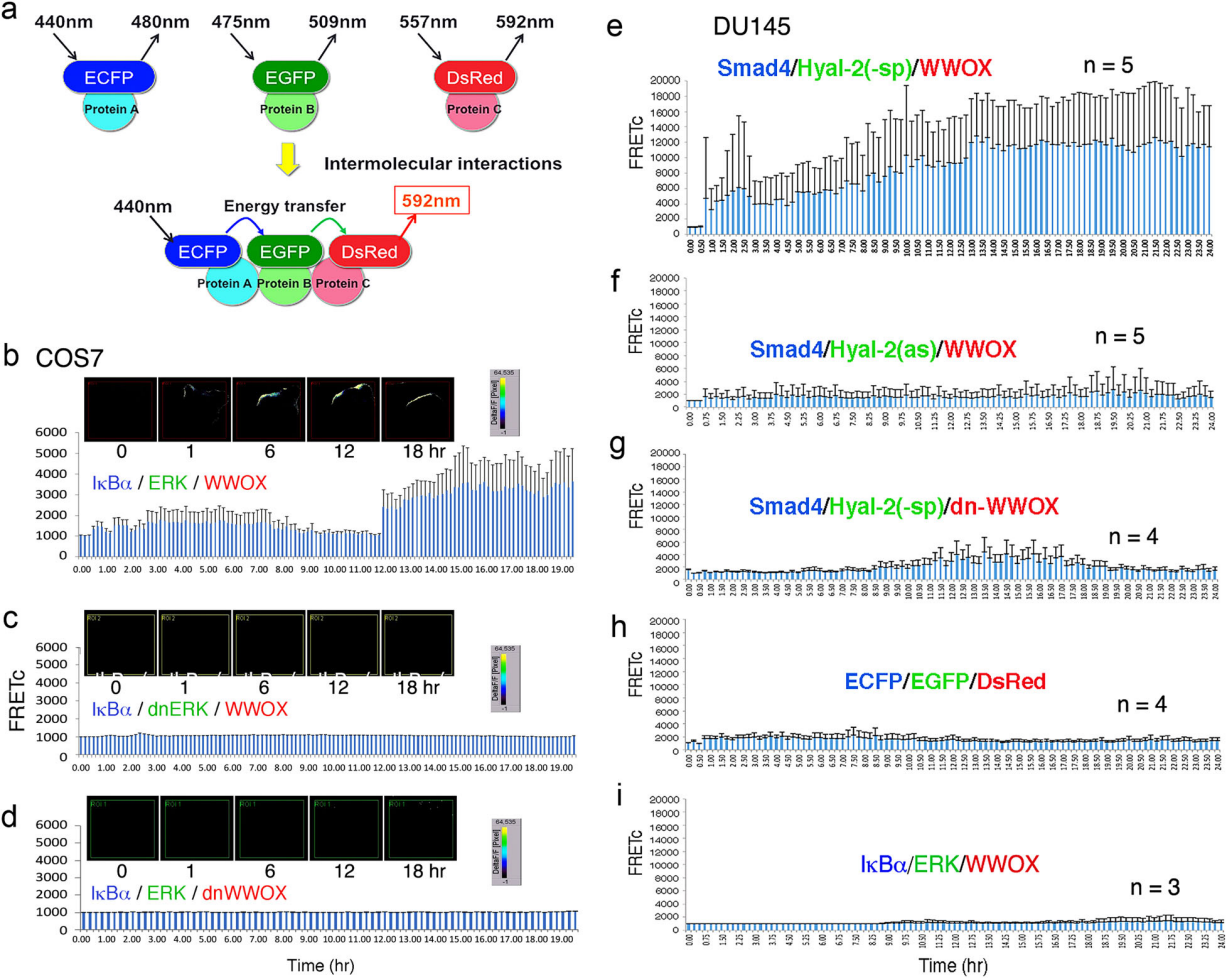
HA initiates Hyal-2/WWOX/Smad4 signaling for bubbling cell death. **a** A depicted graph for the designed tri-molecular FRET analysis is shown. Energy is transferred from ECFP to EGFP and then to DsRed. Briefly, cells were stimulated with an excitation wavelength of 440 nm and emission at 480 nm, which was received by EGFP. EGFP transfers the energy from excitation at 475 nm to emission at 509 nm, which activates DsRed (excitation at 557 nm and emission at 592 nm). FRET signals are detected at an emission wavelength of 592 nm. The FRET images were corrected for background fluorescence from an area free of cells. The spectrally corrected FRET concentration (FRETc) was calculated using an internal software program in the Olympus machine^19,20^. **b** COS7 cells were transiently overexpressed with ECFP-IκBα, EGFP-ERK, and DsRed-monomer WWOX and exposed to IoP for time-lapse FRET microscopy^13^. No cell death occurred. The bar graph is an average of two experiments and its range. The FRET images were corrected for background fluorescence^13–18^. The spectrally corrected FRET concentration (FRETc) was calculated^13–18^. **c**, **d** In negative controls, cells were transiently overexpressed with an indicated dominant-negative EGFP-dnERK or DsRed-monomer dnWWOX (mean ± SD, *n* = 3)^13^. Also, see additional controls using ECFP, EGFP, and/or DsRed vectors only in the Supplemental Data. **e**–**g** DU145 cells were transiently transfected with ECFP-Smd4, EGFP-Hyal-2(-sp) (for cytosolic expression), EGFP-Hyal-2(as) for antisense mRNA, and DsRed-monomer-WWOX expression constructs. HA/Hyal-2 initiates the signaling from the cell surface for the complex formation of ectopic Smad4, Hyal-2, and WWOX^14^. **h**, **i** HA did not induce complex formation of ECFP/EGFP/DsRed and IκBα/ERK/WWOX in DU145 cells^14^ (data in **b**−**d** adapted from ref. ^13^ with modifications and permissions from *J. Biol. Chem*.; **e**−**i** adapted from ref. ^14^ with permissions from *Oncotarget*)

To understand how a molecular signaling path goes and whether the signaling works properly in cells, it is important to measure the protein/protein interactions in vivo in a real-time mode^1–3^. However, many studies regarding the chemistry of protein/protein binding interactions are mainly carried out in vitro^2,3^. End-point measurement is a routine approach to observe the final biological consequences, but it fails to provide the real-time event driving to the end in vivo^4–8^. Förster resonance energy transfer (FRET) microscopy has been widely utilized to determine the inter-structural motif interactions for conformational changes in a single DNA or protein molecule. The molecule is labeled with a donor fluorophore (e.g. cyan fluorescence) and an acceptor fluorophore (e.g. yellow fluorescence). Following excitation, energy from the donor emission excites the acceptor to emit at a lower energy. Also, when both proteins are at a close proximity at nanometer distance (1–10 nm), FRET can be designed to measure two protein-binding interactions^9–12^. Several multiplex-FRET assays have been developed^9–12^. End point assays were mainly carried out in these reports. In brief, for tri-molecular interactions, a pair of donor−acceptor proteins initiates the energy transfer, and the acceptor protein then turns into a donor to transfer the energy to another acceptor protein (Fig. 1a). The energy transfer strategy is designed so that emitted energy from the first protein does not go directly to the third one, as there is no overlap in the emission and the excitation energy range. This can be readily assessed under a microscope with a FRET software program. Additionally, the first donor protein can be designed to simultaneously transfer the energy to two acceptors tagged with different fluorophores for receiving different levels of energy. This approach is good for determining parallel signaling paths from a single starter^9^.

Time-lapse FRET effectively reveals certain biological events. Current knowledge regarding the measure of the dynamics of three-way protein/protein interactions by “time-lapse” FRET microscopy is largely lacking in the literature^9^. By FRET microscopy, we have visualized crucial molecular binding events during cell differentiation, signaling flow, growth and/or death^13–18^. Supporting evidence as shown below reveals that time-lapse tri-molecular FRET microscopy allows determination of the complex formation of the first two molecules as a “driver” leading to the tri-molecular interactions for signaling “execution” and eventual biological outcome. This correlates well with the outcome using routine biochemical and immunological assays. In addition, our assay allows the design for signal branching from a string of straight-line starters of 2–5 proteins or more in the relay.

### IκBα/ERK/WWOX signaling in T-cell maturation visualized by time-lapse FRET microscopy

For time-lapse FRET microscopy^13,14^, an Olympus microscope plus internal software program for FRET has been used^19,20^. In addition to FRET, we have validated the protein binding interactions by co-immunoprecipitation, yeast two-hybrid analysis, confocal co-localization, and co-localization by immunoelectron microscopy^13–18,21^. As simple examples, in our recent reports^13,14^, we have deciphered the signaling path by measuring tri-molecular binding interactions using time-lapse FRET microscopy. By using calcium ionophore A23187 and phorbol myristate acetate (IoP), Huang et al. demonstrated that the underlying IκBα/WWOX/ERK signaling is involved in forced differentiation of acute lymphoblastic leukemia MOLT-4 T cells^13,14,21–23^. As a tumor suppressor^21–23^, endogenous Tyr33-phosphorylated WW domain-containing oxidoreductase (WWOX) binds the non-PEST area of inhibitor of nuclear factor κB (IκBα) and extracellular signal-regulated kinases (ERK) in MOLT-4, as determined by co-immunoprecipitation, yeast two-hybrid analysis, and end-point FRET microscopy^13^. IoP rapidly causes WWOX dephosphorylation at Tyr33 and Tyr287 and phosphorylation at Ser14 in 1–2 h, which leads to phosphorylation of ERK and IκBα in the complex. The Ser14-phosphorylated WWOX appears to play a key role in deciding the downstream signaling for cell maturation^13^. In the next 3–12 h, proteosomal degradation of p-IκBα occurs due to polyubiquitination and ERK de-phosphorylation continues to occur. Later, a portion of WWOX and ERK re-associates and undergoes nuclear accumulation, so as to induce the expression of T-cell maturation antigens CD3 and CD8 in 15–24 h^13^. Specific inhibition of ERK phosphorylation by U0126 or control of IκBα degradation by MG132 abolishes the MOLT-4 maturation^13^.

In parallel experiments, we made mammalian cDNA expression constructs of ECFP-IκBα, EGFP-ERK, and DsRed-monomer WWOX, and showed that IoP-induced FRET energy transfer starts from ECFP-IκBα to EGFP-ERK and then to DsRed-monomer WWOX (Fig. 1b–d)^13^. The data shows that a significant increase in the complex formation of ectopic IκBα/ERK/WWOX occurs just prior to the maturation of T cells in 12–13 h and lasts for more than 20 h^13^. Without IoP treatment, no signals are observed (data not shown). In empty vector controls, no emission energy is shown for ECFP/EGFP/DsRed and ECFP/DsRed (Fig. S1 in the Supplemental Material). There is no overlap in the emission wavelength for ECFP and exciting wavelength for DsRed^24^. The FRET signals can be directly visualized under the microscope with the Olympus FRET analysis program^19,20^. The emitted energy from ECFP is relayed through EGFP to DsRed. Normalized FRET signals were obtained by correcting background fluorescence from an area free of cells and spectral bleed-through^19,20^. No apparent self-oligomerization or functional alteration for IκBα, ERK, and WWOX occurs due to fluorescent tags, or tags used in yeast two-hybrid analysis^13,14^. EGFP or ECFP-tagged IκBα, ERK and WWOX proteins, for example, can be used for co-immunoprecipitation in domain/domain binding interactions^13^.

The reason for choosing ECFP/EGFP/DsRed as interacting partners, rather than ECFP/EYFP/DsRed, is that there is a broad overlapping range between the emission for EYFP and the excitation for DsRed. This will result in significant non-specific signals. Time-lapse FRET microscopy was carried out in COS7 cells, rather than MOLT-4 cells, simply because of their large sizes (Fig. 1b–d), easy transfection with cDNA constructs for expression, and adherence to plastic plates in nature. Nonetheless, both COS7 and MOLT-4 cells are responsive to IoP to induce the IκBα/ERK/WWOX signaling^13^.

### Hyaluronan initiates Hyal-2/WWOX/Smad4 signaling for bubbling cell death

**Fig. 2:**
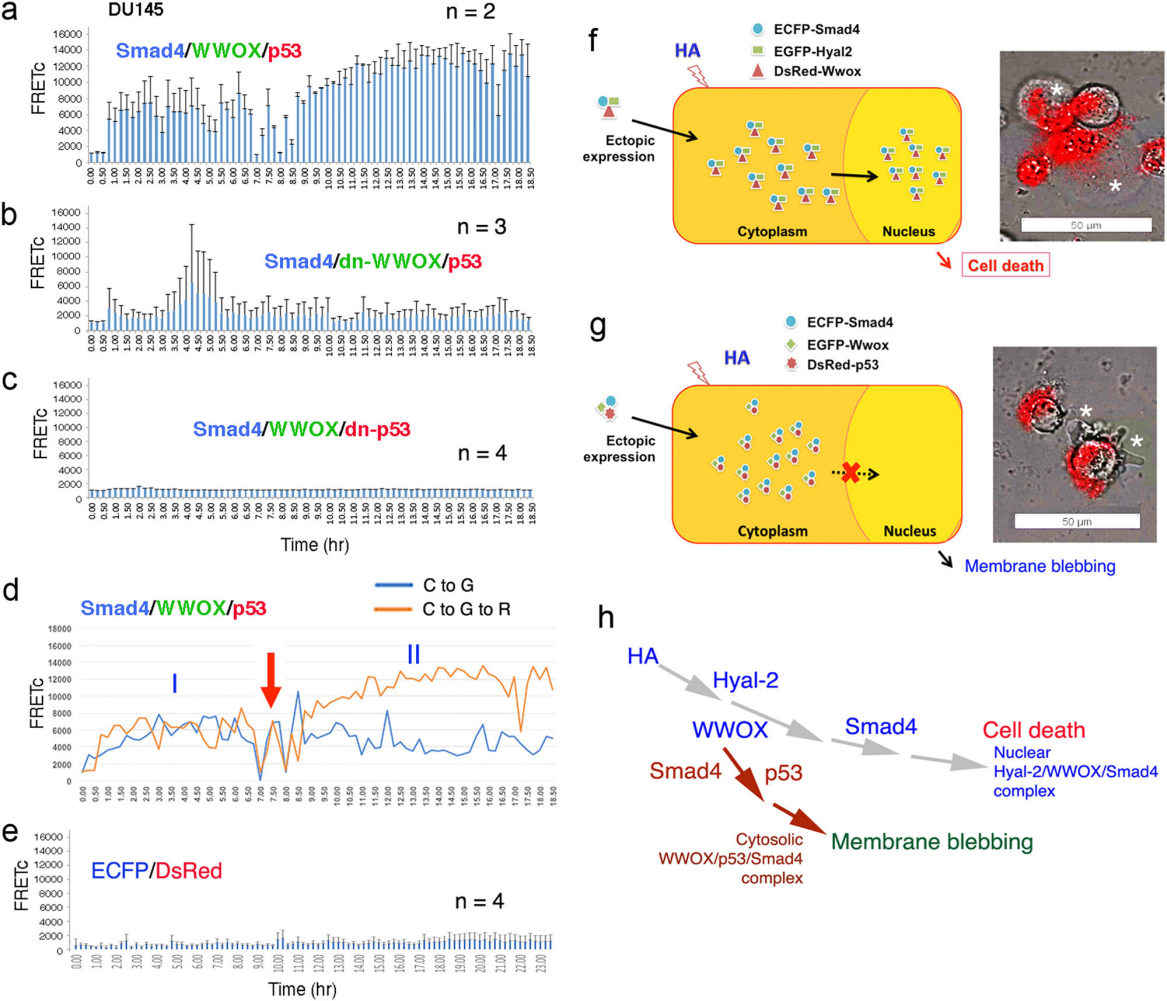
HA initiates alternative p53/WWOX/Smad4 signaling for membrane blebbing. **a** DU145 cells were transiently transfected with ECFP-Smad4, EGFP-WWOX, and DsRed-monomer-p53 expression constructs. HA stimulates membrane Hyal-2 signaling for the complex formation of ectopic Smad4, WWOX, and p53. **b**, **c** Dominant-negative WWOX and p53 abolish the signaling. **d** FRETc signals are measured for cyan to green fluorescence and cyan to green and then to red fluorescence, respectively, which reveals upregulation of Smad4/WWOX/p53 complex formation after treatment with HA for more than 7 h. There are two phases of molecular interactions (see the red arrow separating the phases). In the phase I, Smad4 binds WWOX, and in the phase II, p53 joins the Smad4/WWOX complex. **e** In negative controls, HA-mediated ECFP/DsRed signaling is shown. **f**, **g** Schematic graphs for Smad4/Hyal-2/WWOX-induced bubbling cell death (white stars), and Smad4/WWOX/p53-induced membrane blebbling (white stars) without cell death. **h** Signal branching is depicted. HA initiates ectopic p53/WWOX/Smad4 signaling for membrane blebbing^14^. When the ectopic p53 is replaced with Hyal-2(-sp), HA/Hyal-2 signals the formation of the ectopic Smd4/Hyal-2(-sp)/WWOX complex to cause bubbling cell death^14^ (data in **a**−**c**, **f**, **g** adapted from ref. ^14^ with modifications and permissions from *Oncotarget*)

To further demonstrate the validity of time-lapse FRET for signaling, here we show hyaluronan (HA)-mediated cell death caused by transiently overexpressed proteins^14^. We have determined that Hyal-2/WWOX/Smad4 signaling is involved in traumatic brain injury in rats^14,21,23^. Accumulation of the Hyal-2/WWOX/Smad4 signaling complex in the nucleus causes neuronal death, due to over-activation of the SMAD responsive element^21,23^. Hyaluronidase Hyal-2 is a membrane-anchored protein and a cognate receptor for HA and transforming growth factor beta (TGF-β)^21^. Prostate DU145 cells are transfected with cDNA expression constructs for Smad4, WWOX and Hyal-2(-sp) and then treated with HA^14^. Hyal-2(-sp) cDNA is for cytosolic expression due to lack of membrane glycosylphosphatidylinositol linkage. Membrane Hyal-2 receptor is functional in DU145. HA continuously induces the complex formation of ectopic Smad4, WWOX, Hyal-2(-sp) in the cytoplasm and the complex relocates to the nucleus to induce bubbling cell death (Figs. 1e, 2f; Video S4)^14,22,23^. We have recently discovered “bubbling cell death”, which has been defined as formation of a single bubble from the nucleus per cell and release of this swelling bubble from the cell surface to extracellular space that causes cell death^22,25^. As long as the nuclear bubble is generated and released, this irreversibly leads to cell death. Unlike apoptosis, bubbling cell death is not involved in caspase activation and DNA fragmentation^22,25^. Also, unlike necroptosis and apoptosis, bubbling cell death does not exhibit loss of mitochondrial permeability^22,25^. Both antisense Hyal-2 and dominant-negative WWOX block HA-induced complex formation of ectopic Smad4/Hyal2(-sp)/WWOX (Fig. 1f, g; Video S4). HA does not induce the complex formation of ECFP/EGFP/DsRed, ECFP/DsRed, and IκBα/ERK/WWOX (Fig. 1h, i, S2). That is, HA/Hyal-2 signaling does not relay to the IκBα/ERK/WWOX pathway.

The three-color time-lapse FRET assay is good for measuring bubbling cell death, but cannot be used for directly measuring apoptosis. An alternative approach is transfection of cells with EGFP-tagged Smad4, Hyal2(-sp), and WWOX cDNA expression constructs. During imaging, both DAPI and propidium iodide (PI) are added to the cultured cells. Stressed cells normally pick up DAPI first in less than 2 h due to increased nuclear permeability (Wang et al., submitted). Later, these cells are dying and uptake PI. PI is a stain for measuring cell death^26^.

### HA initiates alternative p53/WWOX/Smad4 signaling for causing membrane blebbing

Many single signal pathways are diverted into branches in the downstream, or are converged from different adaptors in the upstream into a single path toward the end. For example, in the signaling of RAS/MEK/ERK^27^, ERK physically interacts with adaptor proteins such as BIM, MCL1, RSK and many others in the downstream^28^. Notably, if ectopic p53 is expressed in cells, HA is still able to signal via endogenous Hyal-2 to induce the formation of ectopic p53/WWOX/Smad4 complex. Without undergoing nuclear accumulation, the p53/WWOX/Smad4 complex causes membrane blebbing but fails to cause cell death (Fig. 2a, g; Video S5)^14^. Both dominant-negative WWOX and p53 block the signaling (Fig. 2b, c)^14^. Transiently overexpressed WWOX is known to block nuclear translocation of many endogenous proteins^21–23,25,29,30^. Failure of p53/WWOX/Smad4 in undergoing nuclear translocation is probably due to the effect of WWOX.

Further analysis of the signal transfer for ECFP/EGFP vs. ECFP/EGFP/DsRed shows an initial increase in the complex formation for both Smad4/WWOX and Smad4/WWOX/p53 with a similar kinetics in less than 7 h (Fig. 2a, d). Later in 7−20 h, a dramatic increase in the formation of the Smad4/WWOX/p53 complex occurs, while the Smad4/WWOX complex formation stays at a basal line. Accumulation of the Smad4/WWOX/p53 complex correlates with the occurrence of membrane blebbing (Fig. 2f, g)^14^, suggesting that p53 actively joins the Smad4/WWOX complex to cause membrane blebbing. The observations suggest that there are two phases of molecular interactions (Fig. 2a, d). In the phase I, Smad4 binds WWOX, and in the phase II, p53 joins the Smad4/WWOX complex. How Smad4/WWOX/p53 induces membrane blebbing remains to be established.

Together, membrane HA/Hyal-2 initiates a sequential assembly of the ectopic Hyal-2(-sp)/WWOX/Smad4 complex for inducing bubbling cell death (Figs. 1e, 2f–h; Video 4)^14^. When ectopic Hyal-2(-sp) is replaced with p53, HA/Hyal-2 signals the formation of the Smd4/WWOX/p53 complex to cause membrane blebbing (Fig. 2f–h; Video 5). No cell death occurs.

### An initial driving or priming force leads to the key signaling event

**Fig. 3:**
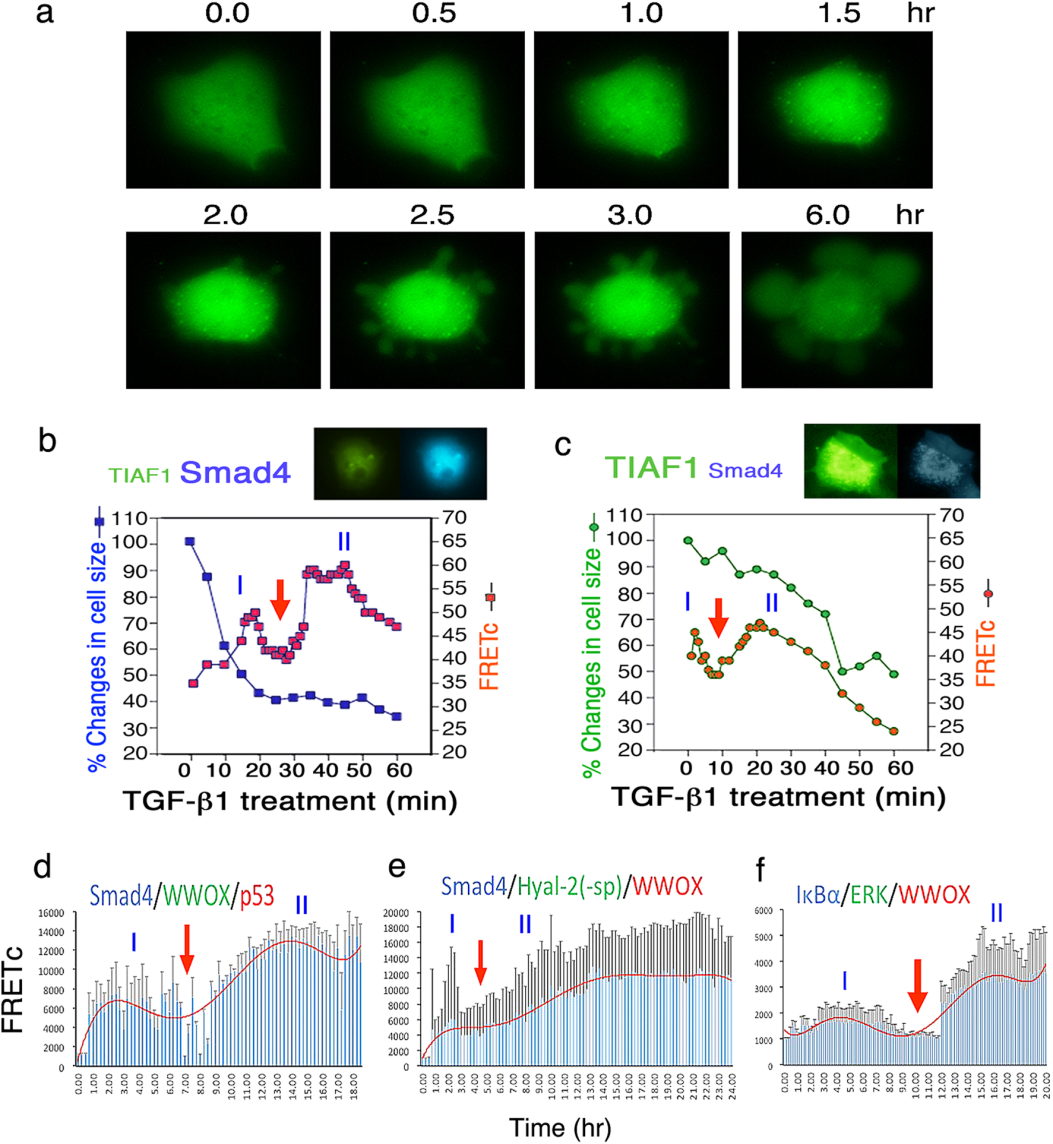
Smad4 overrides TIAF1 self-binding for leading to cell death. **a** TGF-*β*1 (5 ng/ml) induces EYFP-TIAF1 self-aggregation (green punctate) in less an hour, and this leads to apoptosis of NCI-H1299 cells. Also, see Supplemental Video S6. **b** When ECFP-Smad4 is transiently overexpressed at a greater level than EYFP-TIAF1 in NCI-H1299 cells, TGF-*β*1 rapidly increases the binding of Smad4 with TIAF1 in 20 min (Driver, Phase I), which strongly leads to cell death (Execution, Phase II)^18^. **c** In contrast, when the expressed EYFP-TIAF1 level is higher than ECFP-Smad4, cells become refractory to TGF-*β*1-mediated apoptosis. Note that the phase I is within the 10 min range, and FRETc in the phase II is weak^18^. **d**–**f** Exponential regression analysis was carried out for Figs. 2a, 1b, e by Excel software program. Phases I and II are shown. (data in **a**−**c** adapted from ref.^18^ with modifications and permissions from *Cell Death & Disease*)

As mentioned above (Fig. 2d), there is an initial increase in FRETc signal (designated as Driver, Phase I), followed by a brief reduction and then dramatic increase in signal strength by 1−3 fold (Execution, Phase II). One explanation is that the initial accumulation of the bi-molecular complex is needed to “drive” to the tri-molecular interactions of the key signal pathway. In our previous report^18^, we determined that TGF-β1 induces self-polymerization of transiently overexpressed TIAF1 (TGF-β1-induced antiapoptotic factor) for causing cell death (Fig. 3b; Video 6), such as neuronal death in Alzheimer’s disease^18^. That is, self-aggregation occurs prior to apoptosis (Fig. 3a; Video 6). If TIAF1 aggregation is blocked by a dominant-negative or an antisense expression construct, no apoptosis occurs^18^.

Smad4 of the TGF-β signal pathway functionally counteracts with TIAF1^18,31^. Smad4 binds TIAF1 to block its self-polymerization. If ectopic Smad4 is expressed at a higher level than TIAF1, Smad4 suppresses TIAF1 function and apoptosis is accelerated (Fig. 3b)^18^. By FRET analysis, a strong driver signal at the phase I is shown and then proceeds to cell death at the phase II. However, if transiently overexpressed TIAF1 is at a greater amount than Smad4, TIAF1 blocks Smad4-mediated cell death (Fig. 3c)^18^. In this case, the driver signal is weak (Fig. 3c)^18^.

By exponential regression analysis for data shown in Fig. 2a (also see Video S5), Smad4/WWOX/p53-regulated membrane blebbing requires the driver signal to jumpstart the execution phase II for membrane blebbing (Fig. 3d). If the driver signal is too strong in the beginning, the driver phase is directly merged with the execution phase such as in the Smad4/Hyal-2(-sp)/WWOX-mediated bubbling cell death (Figs. 1e, 3e; Video S4). In contrast, IoP-induced driver signal is weak (below 2000 in FRETc) and lengthy, and the execution phase does not cause cell death, but rather induces cell differentiation (Figs. 1b, 3f; Video S1)^13^. The aforementioned observations have also been shown in imaging cell death-regulated autofluorescence. That is, there must have a strong driver force to jumpstart the execution (Chang et al., unpublished).

### Perspectives

In summary, we established a real time mode for determining whether an indicated membrane receptor is functional for binding a specific ligand and then recruiting the downstream adaptor proteins. Our design of tri-molecular FRET analysis provides a feasible assessment for the flow of a specific initiating signal and downstream adaptors in the signal pathway by direct visualization. Indeed, this application can facilitate analysis for the signal-induced cell differentiation, migration, cell−cell interaction, and/or apoptosis^13,14,21–23^.

In general, we have shown in the test models that there is a driver or an initiation phase that leads to the execution phase. Interestingly, there is a gap phase with low FRETc between these two phases, which lasts 1–2 h or less. The driver phase may represent the first two-protein binding such as ECFP-Smad4 and EGFP-WWOX, and the execution shows the association of a third protein to the aforementioned complex, i.e. DsRed-p53 to the ECFP-Smad4/EGFP-WWOX. If the driver phase exhibits a low (barely above the background) and prolonged FRETc, cells appear to carry out a differentiation process^13^. However, if the driver has a high FRETc, it drives quickly to merge with the execution phase to undergo apoptosis. The nature of the gap phase is unknown and remains to be established.

For the endogenous HA/Hyal-2 signaling, WWOX and Smad4 are recruited to interact with the membrane Hyal-2 upon HA stimulation. The resulting Hyal-2/WWOX/Smad4 complex undergoes nuclear translocation to regulate cell survival or death, depending upon the strength of the signal^14,21,23^. If SMAD-dependent promoter is overly activated, cells undergo apoptosis^21^. However, when ectopic WWOX, Smad4 and Hyal-2 are transiently overexpressed in the cytoplasm, HA/Hyal-2 signaling induces complex formation of these ectopic proteins to cause bubbling cell death^14,22,25^. The Hyal-2/WWOX/Smad4 signaling is diverted, if p53 is recruited to replace Hyal-2 in the complex^14^. The ectopic p53/WWOX/Smad4 complex is shown to cause membrane blebbing without cell death, arguing that membrane blebbing is an essential step toward apoptosis.

Regarding IoP-induced cell differentiation, IoP directly modulates the formation and dissociation of the endogenous IκBα/ERK/WWOX signaling complex in a time-related manner^13^. The kinetics of complex formation can be visualized by time-lapse FRET microscopy, in which the event corresponds nicely with the status of WWOX phosphorylation and interaction with IκBα and ERK. Ser14-phosphorylated WWOX appears to be most important in driving the signaling event during forced T leukemia cell maturation^13^. IκBα is a potent inhibitor to control the activation or nuclear accumulation of NF-κB. Presumably, the complex formation of IκBα/ERK/WWOX probably breaks apart the IκBα/NF-κB association, and the released NF-κB undergoes nuclear translocation to enhance cell survival and maturation^13^.

Most recently, we have shown that the status of endogenous WWOX phosphorylation determines cell fate, either differentiation or death^32^. For example, when endogenous pY33-WWOX is overly expressed under stress conditions, it carries out apoptosis to eliminate damaged cells^32^. However, if pS14-WWOX is overly expressed, it may represent the status of cancer growth and progression^32,33^, progression of Alzheimer’s disease^34^, and immune cell maturation^13^. The status of differential phosphorylation may provide an explanation as to why Hyal-2/WWOX/Smad4, which has pY33 phosphorylation, induces bubbling cell death^21^. The status of WWOX phosphorylation in Smad4/WWOX/p53, which causes membrane blebbing (with or without committing to cell death), is unknown.

In another instance, when TIAF1 undergoes TGF-β1-mediated self-polymerization, neuronal cells start to die^18,34^. That is, when TIAF1 self-polymerization reaches a plateau as determined by time-lapse FRET (ECFP and EYFP tags), TIAF1-expressing cells undergo apoptosis. This study was intended to address our concern of TIAF1 self-polymerization in the hippocampal and cortical tissues of non-demented human brains at middle ages^18,34^.

In bubbling cell death^22,25^, we have shown many proteins are involved, including NOS2, WWOX, p53, TRAF2, and Hyal-2. These proteins drive cells either toward survival or death in response to UV irradiation and cold shock. We believe that a similar design for signaling measurement by time-lapse FRET microscopy will reveal the underlying mechanism for the formation a single bubble from each cell and its subsequent death. A prospective dual- or tri-molecular complex design for time-lapse FRET can be WWOX/Hyal-2, Smad4/WWOX/Hyal-2, or p53/WWOX/Hyal-2 for causing bubbling cell death^14^ and NOS2/TRAF2/WWOX for enhancing cell survival. TRAF2 binds and counteracts the function of NOS2 and WWOX. These assays allow determination of the key molecules that allow the cells to reach an end-point event of survival or death.

Regarding cell migration, we found out when a cell does not express a specific gene, the knockout cell undergoes retrograde migration upon encountering a wild-type cell (data not shown). To decipher a signal pathway that causes the retrograde migration, similar design can be applied for the time-lapse FRET system. While cell cycle-regulated cell differentiation has largely been determined, we can apply the same approach to determine cell morphological changes (e.g. epithelial−mesenchymal transition).

Finally, signaling from a ligand with a membrane receptor may recruit many adaptors in relaying the signal. Competitive binding among adaptors to the receptor is going to occur. For example, we have determined that Hyal-2 and Smad4 competitively bind WWOX in yeast^14^. That is, the Hyal-2/WWOX/Smad complex is not stable. p53 is a binding partner of WWOX and is able to replace Hyal-2 in the signaling complex and exerts a different signaling outcome^14,21–23^.

## Materials and methods

### Cell lines and cDNA expression constructs

Cell lines used for imaging were monkey kidney COS7 fibroblasts, human prostate DU145 cells, and human lung p53-deficient NCI-H1299 cells. Where indicated, cells were transiently transfected with 2 or 3 cDNA expression constructs, as follows: ECFP-IκBα; ECFP-Smad4; EGFP-ERK; EGFP-WWOX; DsRed-monomer WWOX; DsRed-monomer p53; dominant-negative (dn) EGFP-dnERK; DsRed-monomer dnWWOX; DsRed-monomer dn-p53(S46G); EGFP-Hyal-2(-sp) (for cytosolic expression); EGFP-Hyal-2(as) for antisense mRNA; EYFP-TIAF1^13,14,18^. Control vectors were ECFP, EGFP, EYFP, and DsRed-monomer. Liposome-based GeneFECTOR (Venn Nova, FL, USA) was used for transfecting cells with the DNA expression constructs.

### Time-lapse tri-molecular FRET microscopy

The two-way or three-way protein/protein interaction FRET microscopy was carried out, as described^13,14,18^. FRET analysis was performed using an inverted fluorescence microscope Olympus IX81. For self-binding or two protein-binding interactions, NCI-H1299 cells were transfected with EYFP-TIAF1 alone, followed by stimulating with TGF-β1 (5 ng/ml) to observe the real-time protein aggregation and the extent of cell death^18^. Additionally, cells were transfected with ECFP-Smad4 and EYFP-TIAF1 and then treated with TGF-β1 (5 ng/ml). FRET microscopy was carried out to determine the binding of Smad4 with TIAF1 with time^18^. Excitation wavelength was 440 nm for ECFP, and emission wavelength 535 nm to excite EYFP. That is, ECFP and EYFP were donor and acceptor fluorescent molecules, respectively. Background fluorescence from an area without cells and spectral bleed-through were corrected. The FRET signals were directly visualized under the microscope and quantified as FRET concentration (FRETc) with the Olympus FRET analysis program^19,20^. Similar procedures were designed for three-protein binding in time-lapse FRET microscopy, in which excitation of an ECFP-tagged protein allows excitation of EGFP-tagged target and then DsRed monomer-tagged target (Fig. 1)^13,14^. For cell differentiation signaling, COS7 cells were transfected with ECFP-IκBα, EGFP-ERK, and DsRed-WWOX, and IoP was used to induce the signaling flow^13,14^. In negative control, dn-EGFP-ERK was used to replace the wild-type EGFP-ERK to block the signaling. In addition, for negative controls, binding of ECFP alone with EYFP alone (or ECFP alone with EGFP alone and DsRed monomer alone) was analyzed. The profiles of time-related FRETc changes were analyzed by exponential regression analysis using Microsoft’s Excel software program.

## Electronic supplementary material

Supplemental Material_Kuo et al(PDF 351 kb)

Video S1

Video S2

Video S3

Video S4

Video S5

Video S6
