# Supplementary material for: Chasing the signaling run by tri-molecular time-lapse FRET microscopy
Source: Cell Death Discov. 2018 Mar 22;4:45. doi: 10.1038/s41420-018-0047-4 (PMC5864757; doi:10.1038/s41420-018-0047-4)
Supplement: Supplementary file 1 — Supplemental Material_Kuo et al(PDF 351 kb) [file 41420_2018_47_MOESM1_ESM.pdf]

Supplemental Material

Chasing the signaling run by tri-molecular time-lapse FRET microscopy

Hsiang-Ling Kuo[1], Pei-Chuan Ho[1], Shenq-Shyang Huang[1], Nan-Shan Chang[1,2,3,4,5,*]

[1]Institute of Molecular Medicine, [2]Center of Infectious Disease and Signaling Research, [3]Advanced Optoelectronic Technology Center, National Cheng Kung University, Tainan, Taiwan, ROC; [4]Department of Neurochemistry, New York State Institute for Basic Research in Developmental Disabilities, Staten Island, NY, USA; [5]Graduate Institute of Biomedical Sciences, College of Medicine, China Medical University, Taichung, Taiwan, ROC.

Correspondence to: Nan-Shan Chang, email: changns@mail.ncku.edu.tw

Keywords: Signaling; FRET microscopy; WWOX; Hyal-2; Smad4

Running title: Run, signaling run!

Supplemental Figures

Figure S1. Time-lapse FRET imaging for control vectors. COS7 cells were transiently overexpressed with ECFP and DsRed-monomer (**a**), or ECFP, EGFP and DsRed-monomer (**b**), and exposed to native hyaluronan (HAn) at 25 μg/ml for FRET imaging at 37°C for 24 hr. The signals are at background levels.

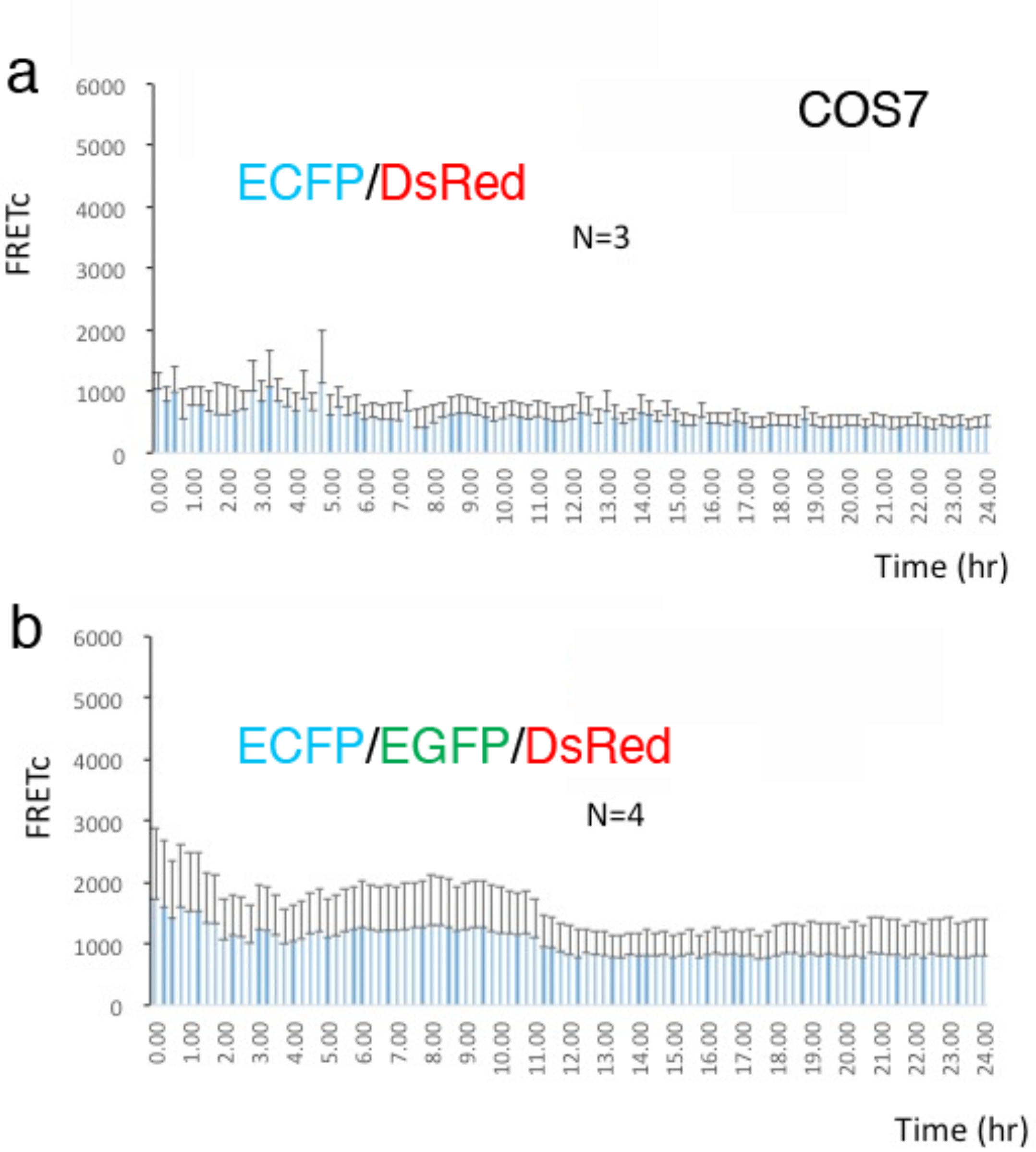

Figure S2. Time-lapse FRET imaging for control vectors. DU145 cells were transiently overexpressed with ECFP and DsRed-monomer (**a**), or ECFP, EGFP and DsRed-monomer (**b**), and exposed to native hyaluronan (HAn) at 25 μg/ml for FRET imaging at 37°C for 24 hr. The signals are at background levels.

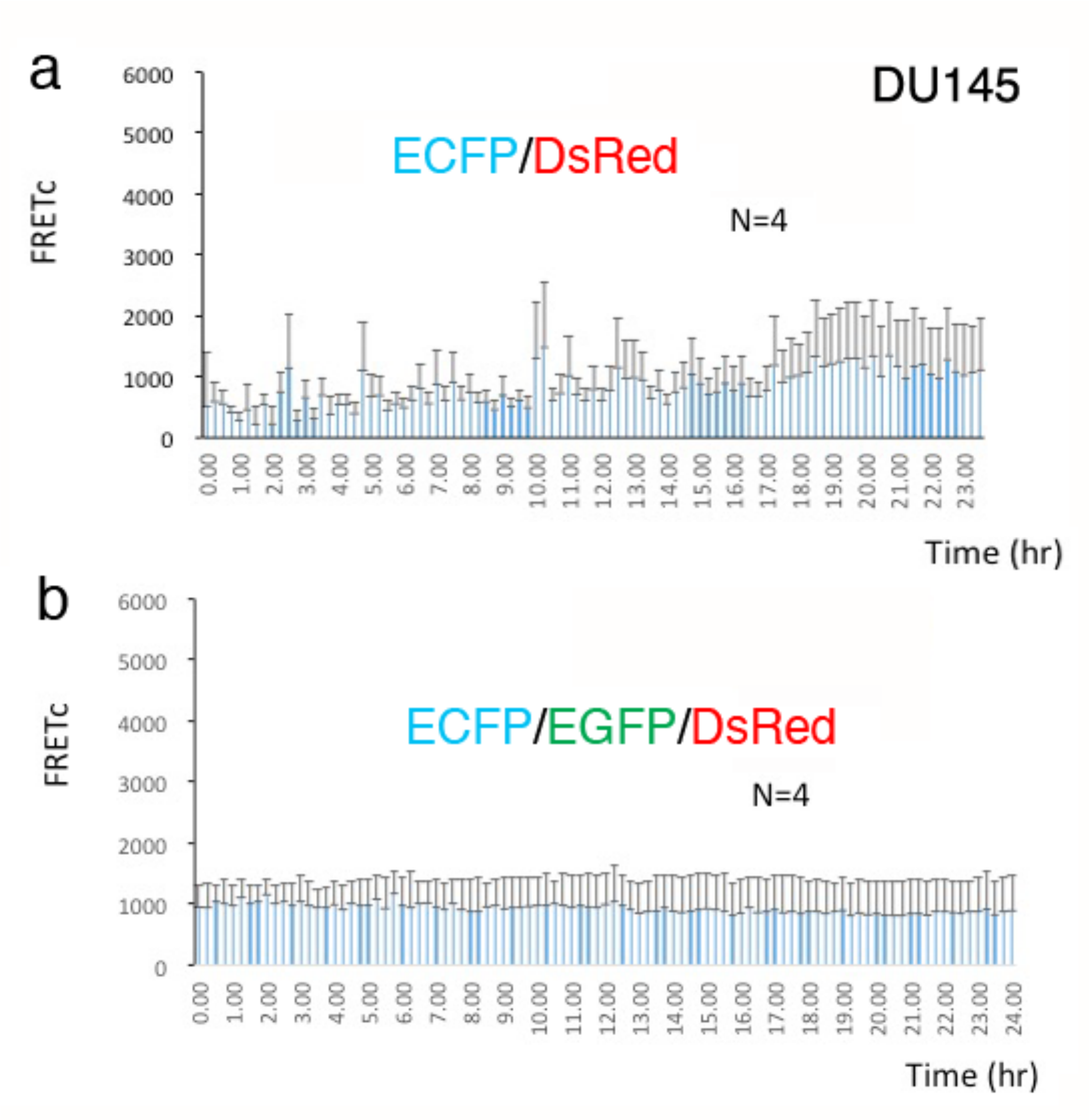

Supplemental Videos

Video S1. Time-lapse FRET microscopy for the binding interactions of ECFP-IκBα, EGFP-ERK and DsRed monomer-WWOX under the influence of IoP in COS7 cells. (adapted from reference #13 with permissions)

Video S2. Time-lapse FRET analysis for the binding interactions of ECFP-IκBα, EGFP-dnERK and DsRed monomer-WWOX under the influence of IoP in COS7 cells. (adapted from reference #13 with permissions)

Video S3. Time-lapse FRET analysis for the binding interactions of ECFP-IκBα, EGFP-ERK and DsRed monomer-dnWWOX under the influence of IoP in COS7 cells. (adapted from reference #13 with permissions)

Video S4. Time-lapse FRET analysis for the Smad4/Hyal-2(-sp)/WWOX signaling in DU145 cells. A merged video of FRETc and bright field is shown. (adapted from reference #14 with permissions)

Video S5. Time-lapse FRET analysis for the Smad4/WWOX/p53 signaling in DU145 cells. A merged video of FRETc and bright field is shown. False positive signals are due to floating dead cells, which may be seen in other videos. (adapted from reference #14 with permissions)

Video S6. TGF-β1-mediated TIAF1 self-aggregation and apoptosis. Lung NCI-H1299 cancer cells were transiently overexpressed with EYFP-TIAF1, followed by stimulating with TGF-β1 (5 ng/ml). By time-lapse microscopy, aggregation of TIAF1 occurred in less than 60 min (see green punctates), followed by membrane blebbing at 180 min. (adapted from reference #31 with permissions)
